# Survival impact of extended cycles of second-line chemotherapy in platinum-sensitive relapsed ovarian cancer patients with residual tumor after six cycles

**DOI:** 10.1186/s12885-020-07658-8

**Published:** 2020-12-07

**Authors:** Se Ik Kim, Woo Yeon Hwang, Maria Lee, Hee Seung Kim, Kidong Kim, Hyun Hoon Chung, Jae Hong No, Jae-Weon Kim, Yong Beom Kim, Noh Hyun Park, Yong-Sang Song, Dong Hoon Suh

**Affiliations:** 1 Department of Obstetrics and Gynecology, Seoul National University College of Medicine, 101 Daehak-Ro Jongno-Gu, Seoul, 03080 Republic of Korea; 2 Department of Obstetrics and Gynecology, Seoul National University Bundang Hospital, 82 Gumi-ro 173 Beon-gil, Bundang-gu, Seongnam-si, Gyeonggi-do 13620 Republic of Korea

**Keywords:** Ovarian cancer, Chemotherapy, Extended, Survival outcome, Recurrence

## Abstract

**Background:**

To determine if extended chemotherapy improves survival outcomes in patients with platinum-sensitive relapsed epithelial ovarian cancer (EOC) who have residual disease after six cycles of second-line chemotherapy.

**Methods:**

In this study, 135 EOC patients who experienced platinum-sensitive recurrence after primary treatment between 2008 and 2018, and had a residual tumor ≥0.5 cm (detected on CT scans) after completing six cycles of second-line, platinum-based chemotherapy, were retrospectively reviewed. Based on the number of main therapy cycles (second-line chemotherapy), we divided patients into an extended group (>6 cycles, *n* = 52) or a standard group (6 cycles, *n* = 83) and compared patient characteristics and survival outcomes between these groups.

**Results:**

The extended group had a shorter platinum-free interval after primary treatment than the standard group (median, 11.0 vs. 13.1 months; *P* = 0.018). Secondary debulking surgery was less frequently performed in the standard group (1.9% vs. 19.3%; *P* = 0.003). After six chemotherapy cycles, the extended and standard groups showed similar serum CA-125 levels (*P* = 0.122) and residual tumor sizes (*P* = 0.232). There was no difference in overall survival (OS) between the groups (*P* = 0.382), although the extended group had significantly worse progression-free survival (PFS) than the standard group (median, 13.9 vs. 15.1 months; *P* = 0.012). Multivariate analyses revealed that platinum-free interval was an independent prognostic factor for PFS and OS, but extended chemotherapy was not (PFS: HR, 1.25; 95% CI, 0.84–1.85; *P* = 0.279; and OS: HR, 1.36; 95% CI, 0.72–2.56; *P* = 0.342). We observed consistent results in the subset of patients who did not undergo secondary debulking surgery.

**Conclusions:**

More than six cycles of platinum-based chemotherapy might not improve survival outcomes in patients with platinum-sensitive recurrent EOC who had a residual tumor ≥0.5 cm after six cycles of second-line chemotherapy.

**Supplementary Information:**

The online version contains supplementary material available at 10.1186/s12885-020-07658-8.

## Background

Epithelial ovarian cancer (EOC) is a gynecologic malignancy and one of the leading causes of death worldwide [1, 2]. Despite initial and subsequent treatments, most patients with advanced EOC experience disease relapse repeatedly because of chemoresistance [3]. Therefore, treating recurrent EOC is challenging. Traditionally, patients with recurrent EOC are categorized into two subsets according to a platinum-free interval (PFI), which is defined as the time between completing the last platinum-based treatment and evidence of disease progression [4]. When the PFI is 6 months or longer, it is called platinum-sensitive relapsed (PSR) EOC, and is known to have a better prognosis than platinum-resistant EOC.

For patients with PSR EOC, current practice guidelines recommend six cycles of platinum-based combination chemotherapy as second-line chemotherapy [5]. To our knowledge, however, no randomized controlled trial (RCT) has been conducted, investigating the optimal cycle numbers of chemotherapy in those settings. The administration of six cycles of second-line chemotherapy seems to be based on the results of phase III RCTs or observational studies in front-line treatment for EOC, which have shown that more than six cycles of conventional cytotoxic chemotherapy do not improve survival outcomes for newly diagnosed EOC patients [6–8]. Nevertheless, more than 6 cycles of platinum-based combination chemotherapy were allowed in monumental phase III RCTs that investigated the efficacy of chemotherapeutic agents in PSR EOC; for example, two (GOG-0213 [9]) or four (OCEANS [10]) additional cycles, and even unlimited until disease progression (CALYPSO [11]).

More than six cycles of chemotherapy might delay the time to disease progression in recurrent EOC. However, there is concern about cumulative toxicity and, subsequently, poor quality of life. Moreover, there is no robust evidence of increased survival from extended chemotherapy [12, 13]. It is difficult to decide how many additional cycles of platinum-based combination chemotherapy would be benefit patients with PSR EOC when the patient has residual tumors despite six cycles of second-line chemotherapy. Thus, we performed this study to determine whether extended cycles of second-line chemotherapy could improve survival outcomes in patients with PSR EOC having residual tumors after six cycles.

## Methods

### Study population

From the two tertiary institutional hospitals’ Ovarian Cancer Cohorts, we included patients with the following conditions: (1) histologically confirmed epithelial ovarian, fallopian tube, or primary peritoneal carcinoma, and (2) relapse ≥6 months after completion of primary treatment consisting of debulking surgery and platinum-based chemotherapy; between 2008 and 2018. Meanwhile, the following patients were excluded: (1) those with other malignancies that might influence survival outcomes, (2) those under active second-line treatment, (3) those who were enrolled in clinical trials, and (4) those who were lost to follow-up or had insufficient clinicopathologic data.

Among patients who met the inclusion criteria described above, we further selected patients based on the number of cycles of the main therapy (platinum-based combination chemotherapy, but not counting maintenance therapy with single agents such as bevacizumab or olaparib) and imaging study results. Patients who had completed at least six cycles of second-line chemotherapy and showed partial response or had stable disease after six cycles of chemotherapy, as evaluated by the Response Evaluation Criteria in Solid Tumors (RECIST) version 1.1 [14], were included in the study. Additionally, only those who had residual tumors (at least 0.5 cm), as confirmed via computed tomography (CT) scans taken within 4 weeks of the sixth chemotherapy cycle, were included. Based on the number of main therapy cycles of second-line chemotherapy, the patients were divided into an extended group (>6 cycles) or a standard group (6 cycles).

### Data collection

We collected clinicopathologic data, such as age, serum CA-125 levels, International Federation of Gynecology and Obstetrics (FIGO) stage, histologic type and grade, and primary treatment details from the patients’ medical records, imaging studies, and pathologic reports. Optimal debulking surgery was considered when the maximum diameter of the residual tumor, after surgery, was less than 1.0 cm. The germline *BRCA1/2* gene test results were also collected. Referring to the five-tier terminology system recommended by the American College of Medical Genetics and Genomics and the Association for Molecular Pathology [15], we regarded “pathogenic” and “likely pathogenic” variants as *BRCA1/2* mutations and other variants as wild-type *BRCA1/2* genes. Details of the second-line treatment were also collected (e.g., secondary debulking surgery, chemotherapy regimen and cycles, and the residual tumor size).

The surveillance methods did not differ between the extended and standard groups. We routinely performed CT scans every three cycles of second-line chemotherapy and every 3 months for the first 2 years, every 4 to 6 months for the next 2 years, and thereafter, every year. Disease progression was assessed according to the RECIST version 1.1 [14]. In terms of survival outcomes, progression-free survival (PFS) and overall survival (OS) were defined as the time intervals between the start of second-line treatment to disease progression and cancer-related death or the end of the study, respectively.

### Statistical analysis

Differences in patient characteristics were evaluated between the extended and standard groups. We used a Student’s t-test or Mann-Whitney U-test to compare continuous variables, and a Pearson’s chi-squared test or Fisher’s exact test to compare categorical variables. For comparison of survival outcomes, we used Kaplan-Meier analysis with a log-rank test. In the multivariate analyses, the hazard ratios (HRs) and 95% confidence intervals (CIs) were calculated using Cox proportional hazards regression models. All analyses were performed using SPSS statistical software (version 25.0; SPSS Inc., Chicago, IL, USA). A value of *P* <0.05 was considered statistically significant.

## Results

Overall, 135 patients were included in this analysis: 52 in the extended group and 83 in the standard group. As an example of residual tumor evaluation using subsequent CT scans, key images from two representative patients are depicted in Supplementary Figure S1.

### Analysis in all patients

Table 1 presents the clinicopathologic characteristics of all patients. No differences in patient age, histologic type, tumor grade, FIGO stage, and primary treatment strategy were observed between the extended and standard groups. In the study population, 84 patients (62.2%) received germline *BRCA1/2* gene testing, and *BRCA1/2* mutational status did not differ between the two groups. Although the proportion of optimal initial debulking surgery was similar (80.8% vs. 79.5%; *P* = 0.860), patients in the extended group achieved complete gross resection less frequently at the time of initial debulking surgery (42.3% vs. 60.2%; *P* = 0.042). At the time of recurrence after primary treatment, the extended group had a significantly shorter PFI (median, 11.0 vs. 13.1 months; *P* = 0.018). However, there was no difference in serum CA-125 levels (median, 99.1 vs. 91.0 IU/mL; *P* = 0.152).

**Table 1:** Clinicopathologic characteristics of study population

Characteristics	*All* (*n* = 135)	*Extended chemotherapy* (*n* = 52)	*Standard chemotherapy* (*n* = 83)	*P*
Age at initial diagnosis, years	55.5 ± 10.1	55.0 ± 9.0	55.9 ± 10.7	0.624
Age at 1st recurrence, years	57.4 ± 9.9	56.6 ± 9.1	57.9 ± 10.4	0.475
Primary site of disease				0.314
Ovary	123 (91.1)	46 (88.5)	77 (92.8)	
Tube	4 (3.0)	1 (1.9)	3 (3.6)	
Peritoneum	8 (5.9)	5 (9.6)	3 (3.6)	
Histologic type				0.344
Serous	120 (88.9)	46 (88.5)	74 (89.2)	
Endometrioid	8 (5.9)	3 (5.8)	5 (6.0)	
Mucinous	1 (0.7)	1 (1.9)	0	
Clear cell	3 (2.2)	2 (3.8)	1 (1.2)	
Mixed	3 (2.2)	0	3 (3.6)	
Grade				0.136
1	4 (3.0)	3 (5.8)	1 (1.2)	
2	7 (5.2)	1 (1.9)	6 (7.2)	
3	124 (91.9)	48 (92.3)	76 (91.6)	
FIGO stage				0.496
I-II	9 (6.7)	5 (9.6)	4 (4.8)	
III	88 (65.2)	34 (65.4)	54 (6.1)	
IV	38 (28.1)	13 (25.0)	25 (30.1)	
Primary treatment strategy				0.540
PDS	100 (74.1)	37 (71.2)	63 (75.9)	
NAC	35 (25.9)	15 (28.8)	20 (24.1)	
Results of initial debulking surgery				0.041
Complete gross resection	72 (53.3)	22 (42.3)	50 (60.2)	
Residual tumor <1 cm	36 (26.7)	20 (38.5)	16 (19.3)	
Residual tumor ≥1 cm	27 (20.0)	10 (19.2)	17 (20.5)	
Platinum-free interval, months				
Median (range)	12.5 (6.0–87.9)	11.0 (6.2–87.9)	13.1 (6.0–84.5)	0.018
6–12, partially platinum-sensitive	61 (45.2)	28 (53.8)	33 (39.8)	0.109
≥12, totally platinum-sensitive	74 (54.8)	24 (46.2)	50 (60.2)	
CA-125 at 1st recurrence, IU/mL				0.152
Median (range)	91.0 (7.9–6290.0)	99.1 (12.0–6290.0)	91.0 (7.9–1908.0)	
Secondary debulking surgery				0.003
No	118 (87.4)	51 (98.1)	67 (80.7)	
Yes	17 (12.6)	1 (1.9)	16 (19.3)	
Results of secondary debulking surgery				0.279
Complete gross resection	6 (4.4)	0	6 (7.2)	
Residual tumor <1 cm	6 (4.4)	0	6 (7.2)	
Residual tumor ≥1 cm	5 (3.7)	1 (1.9)	4 (4.8)	
Residual tumor on CT after #6				0.232
≥0.5 cm and <1 cm	65 (48.1)	28 (53.8)	37 (44.6)	
≥1 cm and <2 cm	56 (41.5)	17 (32.7)	39 (47.0)	
≥2 cm	14 (10.4)	7 (13.5)	7 (8.4)	
CA-125 after #6, IU/mL				0.122
Median (range)	12.5 (1.1–445.0)	14.0 (1.1–249.0)	11.0 (1.1–445.0)	
Maintenance therapy				
No	98 (72.6)	46 (88.5)	52 (62.7)	0.001
Yes	37 (27.4)	6 (11.5)	31 (37.3)	
Bevacizumab	29 (21.5)	5 (9.6)	24 (28.9)	>0.999
Olaparib	8 (5.9)	1 (1.9)	7 (8.4)	
Germline *BRCA1/2* gene test				
Not performed	51 (37.8)	23 (44.2)	28 (33.7)	0.221
Performed	84 (62.2)	29 (55.8)	55 (66.3)	
Wild-type	58 (43.0)	21 (40.4)	37 (44.6)	0.628
Mutation	26 (19.3)	8 (15.4)	18 (21.7)	

Values are presented as mean ± standard deviation or n (%) unless otherwise indicated

*Abbreviations*: *CA-125* Cancer antigen 125, *CT* Computed tomography, *FIGO* International Federation of Gynecology and Obstetrics

In terms of second-line treatment, 17 patients underwent secondary debulking surgery followed by chemotherapy, while 118 patients received second-line chemotherapy only. Secondary debulking surgery was performed less often in the extended group than in the standard group (1.9% vs. 19.3%; *P* = 0.003). However, after six cycles of second-line chemotherapy, there were no differences in serum CA-125 levels (*P* = 0.122) and residual tumor sizes (*P* = 0.232), between the two groups (Table 1).

Details of the second-line treatment are shown in Supplementary Table S1. In the extended group, the median main therapy cycle number of second-line chemotherapy was 9 (range, 7–15), and the most common chemotherapy regimen was paclitaxel plus carboplatin (*n* = 23, 44.2%). Of the 52 patients, 6 (11.5%) received maintenance therapy after chemotherapy: 5 (9.6%) received bevacizumab maintenance, and 1 (1.9%) received olaparib maintenance. In the standard group, the most common chemotherapy regimen was paclitaxel plus carboplatin (*n* = 35, 42.2%), followed by paclitaxel plus carboplatin with bevacizumab (*n* = 24, 28.9%) and pegylated liposomal doxorubicin plus carboplatin (*n* = 12, 14.5%). Of the 83 patients, 31 (37.3%) received maintenance therapy after six cycles of chemotherapy: 24 (28.9%) received bevacizumab maintenance, and 7 (8.4%) received olaparib maintenance.

**Fig. 1:**
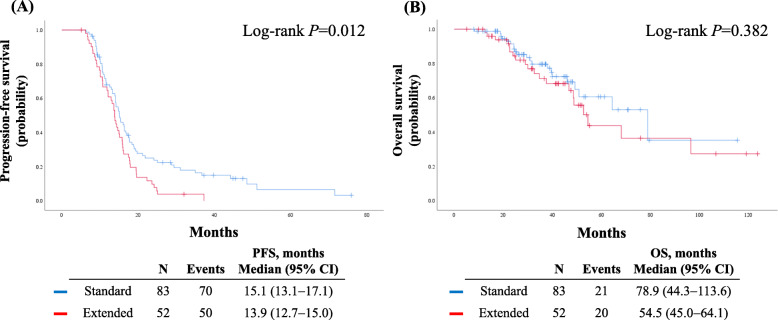
Comparisons of survival outcomes between the extended and standard chemotherapy groups. **a** Progression-free survival; **b** Overall survival

During a median length of observation of 32.7 months (range, 5.1–123.8 months), 120 patients (88.9%) experienced disease recurrence, and 41 patients (30.4%) expired. Patients in the extended group had significantly worse PFS than those in the standard group (median, 13.9 vs. 15.1 months; *P* = 0.012). However, no significant difference was observed in OS between the two groups (*P* = 0.382) (Fig. 1).

Multivariate analyses adjusting for variables such as PFI and residual tumor size on CT after six cycles of chemotherapy revealed that extended chemotherapy did not influence either PFS (adjusted HR, 1.25; 95% CI, 0.84–1.85; *P* = 0.279) or OS (adjusted HR, 1.36; 95% CI, 0.72–2.56; *P* = 0.342) (Table 2). For PFS, PFI <12 months and higher serum CA-125 levels (≥90 IU/mL) at the first recurrence were identified as poor prognostic factors, whereas maintenance therapy was a favorable prognostic factor. For OS, PFI <12 months and a residual tumor ≥1 cm on CT after 6 cycles of chemotherapy were poor prognostic factors. Meanwhile, higher serum CA-125 levels (≥90 IU/mL) at the first recurrence showed a trend toward worse OS with borderline statistical significance (*P* = 0.050) (Table 2).

**Table 2:** Univariate and multivariate analyses for survival outcomes in whole study population

Characteristics	*Progression-free survival*					*Overall survival*				
	Univariate analysis		Multivariate analysis			Univariate analysis		Multivariate analysis		
	HR	95% CI	aHR	95% CI	*P*	HR	95% CI	aHR	95% CI	*P*
Age at initial diagnosis, years										
≥55 vs. <55	0.83	0.57–1.19	0.75	0.51–1.08	0.122	0.96	0.51–1.78			
Histologic type										
Non-HGSC vs. HGSC	1.10	0.64–1.90				0.80	0.29–2.26			
FIGO stage										
IV vs. I-III	1.15	0.77–1.71	0.81	0.53–1.22	0.308	0.98	0.48–2.00	0.78	0.37–1.66	0.515
Primary treatment strategy										
NAC vs. PDS	0.92	0.60–1.39				2.28	1.20–4.34			
Results of initial debulking surgery										
Residual tumor vs. CGR	1.16	0.81–1.66				1.26	0.68–2.36			
Platinum-free interval, months										
≥12 vs. 6–12	0.46	0.31–0.66	0.42	0.29–0.63	<0.001	0.36	0.19–0.67	0.37	0.19–0.70	0.003
CA-125 at 1st recurrence, IU/mL										
≥90 vs. <90	1.76	1.22–2.53	2.21	1.50–3.25	<0.001	1.92	1.02–3.60	1.90	1.00–3.60	0.050
Secondary debulking surgery										
Yes vs. No	0.67	0.37–1.22				0.88	0.27–2.88			
Residual tumor on CT after #6										
≥1 cm vs. <1 cm	1.09	0.76–1.56	1.20	0.83–1.74	0.326	2.01	1.04–3.90	2.38	1.20–4.71	0.013
CA-125 after #6, IU/mL										
≥12.5 vs. <12.5	1.50	1.04–2.15				1.47	0.79–2.73			
Maintenance therapy										
Yes vs. No	0.50	0.32–0.76	0.44	0.28–0.69	<0.001	0.67	0.30–1.53	0.65	0.28–1.52	0.317
Extended chemotherapy										
Yes vs. No	1.61	1.11–2.34	1.25	0.84–1.85	0.279	1.32	0.71–2.44	1.36	0.72–2.56	0.342

*Abbreviations*: *aHR* Adjusted HR, *CA-125* Cancer antigen 125, *CGR* Complete gross resection, *CI* Confidence interval, *CT* Computed tomography, *FIGO* International Federation of Gynecology and Obstetrics, *HGSC* High-grade serous carcinoma, *HR* Hazard ratio

### Analysis in patients who did not receive secondary debulking surgery

For robust survival analyses, we excluded 17 patients who underwent secondary debulking surgery. The clinicopathologic characteristics of patients in this subgroup are presented in Table 3. Of 118 patients, 69 (58.5%) received germline *BRCA1/2* gene testing, and *BRCA1/2* mutational status did not differ between the extended and standard groups. Among the variables, the initial debulking surgery results were significantly different between the two groups (*P* = 0.036). However, the proportions of optimal initial debulking surgery (80.4% vs. 74.6%; *P* = 0.460) and cases that achieved complete gross resection (41.2% vs. 56.7%; *P* = 0.094) were similar between the extended and standard groups. After six cycles of chemotherapy, no differences in serum CA-125 levels (*P* = 0.087) and residual tumor size, measured by CT scans (*P* = 0.446), were observed between the groups. After main therapy (platinum-based), patients in the extended group underwent significantly less maintenance therapy than those in the standard group (11.8% vs. 32.8%; *P* = 0.008) (Table 3).

**Table 3:** Clinicopathologic characteristics of the patients who did not receive secondary debulking surgery

Characteristics	*All* (*n* = 118)	*Extended chemotherapy* (*n* = 51)	*Standard chemotherapy* (*n* = 67)	*P*
Age at initial diagnosis, years	56.1 ± 10.0	54.8 ± 9.0	57.1 ± 10.6	0.208
Age at 1st recurrence, years	57.9 ± 9.9	56.4 ± 9.0	59.0 ± 10.5	0.159
Primary site of disease				0.499
Ovary	107 (90.7)	45 (88.2)	62 (92.5)	
Tube	3 (2.5)	1 (2.0)	2 (3.0)	
Peritoneum	8 (6.8)	5 (9.8)	3 (4.5)	
Histologic type				0.381
Serous	106 (89.8)	46 (90.2)	60 (89.6)	
Endometrioid	6 (5.1)	3 (5.9)	3 (4.5)	
Mucinous	3 (2.5)	2 (3.9)	1 (1.5)	
Clear cell	3 (2.5)	0	3 (4.5)	
Mixed				
Grade				0.113
1	2 (1.7)	2 (3.9)	0	
2	6 (5.1)	1 (2.0)	5 (7.5)	
3	110 (93.2)	48 (94.1)	62 (92.5)	
FIGO stage				0.686
I-II	7 (5.9)	4 (7.8)	3 (4.5)	
III	78 (66.1)	34 (66.7)	44 (65.7)	
IV	33 (28.0)	13 (25.5)	20 (29.9)	
Primary treatment strategy				0.625
PDS	86 (72.9)	36 (70.6)	50 (74.6)	
NAC	32 (27.1)	15 (29.4)	17 (25.4)	
Results of initial debulking surgery				0.036
Complete gross resection	59 (50.0)	21 (41.2)	38 (56.7)	
Residual tumor <1 cm	32 (27.1)	20 (39.2)	12 (17.9)	
Residual tumor ≥1 cm	27 (22.9)	10 (19.6)	17 (25.4)	
Platinum-free interval, months				
Median (range)	12.2 (6.0–87.9)	11.0 (6.2–87.9)	12.7 (6.0–57.8)	0.059
6–12, partially platinum-sensitive	55 (46.6)	27 (52.9)	28 (41.8)	0.229
≥12, totally platinum-sensitive	63 (53.4)	24 (47.1)	39 (58.2)	
CA-125 at 1st recurrence, IU/mL				0.145
Median (range)	93.3 (7.9–6290.0)	107.9 (12.0–6290.0)	92.9 (7.9–1908.0)	
Residual tumor on CT after #6				0.446
≥0.5 cm and <1 cm	57 (48.3)	27 (52.9)	30 (44.8)	
≥1 cm and <2 cm	47 (39.8)	17 (33.3)	30 (44.8)	
≥2 cm	14 (11.9)	7 (13.7)	7 (10.4)	
CA-125 after #6, IU/mL				0.087
Median (range)	12.7 (1.1–289.7)	14.0 (1.1–249.0)	11.0 (1.1–289.7)	
Maintenance therapy				
No	90 (76.3)	45 (88.2)	45 (67.2)	0.008
Yes	28 (23.7)	6 (11.8)	22 (32.8)	
Bevacizumab	23 (19.5)	5 (9.8)	18 (26.9)	>0.999
Olaparib	5 (4.2)	1 (2.0)	4 (6.0)	
Germline *BRCA1/2* gene test				
Not performed	49 (41.5)	23 (45.1)	26 (38.8)	0.492
Performed	69 (58.5)	28 (54.9)	41 (61.2)	
Wild-type	47 (39.8)	20 (39.2)	27 (40.3)	0.626
Mutation	22 (18.6)	8 (15.7)	14 (20.9)	

Values are presented as mean ± standard deviation or n (%) unless otherwise indicated

*Abbreviations*: *CA-125* Cancer antigen 125, *CT* Computed tomography, *FIGO* International Federation of Gynecology and Obstetrics

**Fig. 2:**
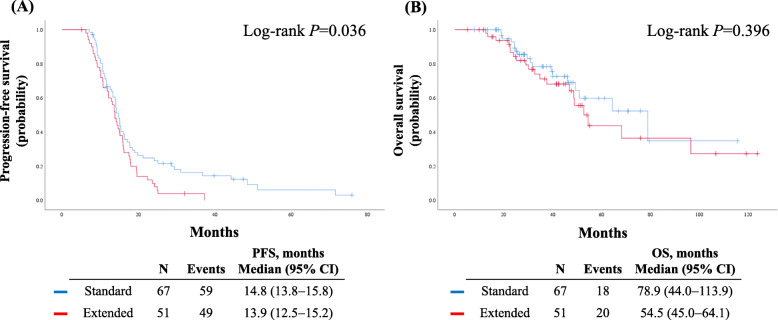
Comparisons of survival outcomes in the subgroup of patients who did not receive secondary debulking surgery. **a** Progression-free survival; **b** Overall survival

During a median length of observation of 33.7 months (range, 5.1–123.8 months), 108 patients (91.5%) experienced disease recurrence, and 38 patients (32.2%) died. Consistent with the results in the whole study population, patients in the extended group showed significantly worse PFS than those in the standard group (median, 13.9 vs. 14.8 months; *P* = 0.036). However, no significant difference was observed in OS between the two groups (*P* = 0.396) (Fig. 2).

In this subgroup, the multivariate analyses also revealed that extended chemotherapy did not influence PFS (adjusted HR, 1.18; 95% CI, 0.78–1.78; *P* = 0.428) or OS (adjusted HR, 1.42; 95% CI, 0.74–2.73; *P* = 0.297) (Table 4). For PFS, PFI <12 months and higher serum CA-125 levels (≥90 IU/mL) at the first recurrence were identified as independent poor prognostic factors, whereas maintenance therapy was a favorable prognostic factor. For OS, PFI <12 months, and a residual tumor ≥1 cm on CT after 6 cycles of chemotherapy were poor prognostic factors (Table 4).

**Table 4:** Univariate and multivariate analyses for survival outcomes in patients who did not receive secondary debulking surgery

Characteristics	*Progression-free survival*					*Overall survival*				
	Univariate analysis		Multivariate analysis			Univariate analysis		Multivariate analysis		
	HR	95% CI	aHR	95% CI	*P*	HR	95% CI	aHR	95% CI	*P*
Age at initial diagnosis, years										
≥55 vs. <55	0.75	0.51–1.11	0.714	0.483–1.057	0.093	0.80	0.42–1.54			
Histologic type										
Non-HGSC vs. HGSC	1.01	0.54–1.91				0.75	0.23–2.45			
FIGO stage										
IV vs. I-III	1.14	0.74–1.73				0.94	0.44–2.00	0.74	0.33–1.64	0.461
Primary treatment strategy										
NAC vs. PDS	1.13	0.74–1.74				2.48	1.28–4.79			
Results of initial debulking surgery										
Residual tumor vs. CGR	1.15	0.79–1.68				1.22	0.63–2.34			
Platinum-free interval, months										
≥12 vs. 6–12	0.44	0.29–0.65	0.40	0.27–0.61	<0.001	0.37	0.19–0.71	0.37	0.19–0.72	0.004
CA-125 at 1st recurrence, IU/mL										
≥90 vs. <90	1.60	1.09–2.35	1.88	1.26–2.82	0.002	1.77	0.92–3.42	1.78	0.91–3.47	0.090
Residual tumor on CT after #6										
≥1 cm vs. <1cm	1.12	0.76–1.63	1.23	0.84–1.81	0.294	1.79	0.91–3.52	2.10	1.05–4.22	0.036
CA-125 after #6, IU/mL										
≥12.5 vs. <12.5	1.58	1.08–2.32	1.18	0.79–1.76	0.412	1.36	0.72–2.58			
Maintenance therapy										
Yes vs. No	0.48	0.30–0.77	0.44	0.26–0.72	0.001	0.69	0.29–1.66	0.71	0.29–1.76	0.456
Extended chemotherapy										
Yes vs. No	1.51	1.02–2.24	1.18	0.78–1.78	0.428	1.32	0.70–2.51	1.42	0.74–2.73	0.297

*Abbreviations*: *aHR* Adjusted HR, *CA-125* Cancer antigen 125, *CGR* Complete gross resection, *CI* Confidence interval, *CT* Computed tomography, *FIGO* International Federation of Gynecology and Obstetrics, *HGSC* High-grade serous carcinoma, *HR* Hazard ratio

## Discussion

In the present study, we evaluated the impact of extended second-line chemotherapy on the survival of patients with PSR EOC who had a residual tumor despite six cycles of chemotherapy. Compared to the standard six cycles, administration of more than six cycles of platinum-based combination chemotherapy did not improve PFS or OS. Consistent results were also observed after the exclusion of patients who underwent secondary debulking surgery.

To date, the exact role of extended platinum-based combination chemotherapy in PSR EOC has not yet been clearly established. However, clinicians tend to recommend extended chemotherapy to patients with the following conditions: widely disseminated, relapsed disease; short PFI; tumors that have not been surgically removed; or lack of complete remission despite the standard six cycles of chemotherapy. As a reflection of such tendencies in real-world clinical practice, some characteristics were different between the two groups in the current study. Compared to the standard group, the extended group had significantly shorter PFI and less commonly had secondary debulking surgery. Despite these differences, the two groups showed similar serum CA-125 levels and residual tumor size measured by CT scans after six second-line chemotherapy cycles. Nevertheless, we adjusted for these factors in subsequent multivariate analyses to control for intrinsic selection bias.

All patients in our study population had a residual tumor ≥0.5 cm as measured by CT scans after six cycles of chemotherapy. The rationale for the extended use of chemotherapeutic agents in these patients was the accumulation of additional cytotoxic or anti-cancer effects on the residual tumors. A previous retrospective study reported the presence of late (>6 cycles of chemotherapy) responders [16]. Carrying out the current study, we expected a benefit in survival from extended chemotherapy. However, contrary to our expectation, multivariate analyses revealed that extended chemotherapy did not influence either PFS or OS. As a hypothesis to explain these results, we attribute the presence of cancer stem cells (CSCs) within residual tumors [17, 18] and tumor evolution towards drug resistance during extended chemotherapy [19, 20]. Some properties of CSCs, such as slow cell cycles, reduced uptake, and increased efflux of drugs, seemed to offset the effect of extended use of conventional chemotherapeutic drugs [21]. Considering these aspects, the concept of increased survival by extending platinum-based combination chemotherapy as long as there are no cumulative toxicities or side effects do not seem reasonable.

Interestingly, our study results demonstrated that a residual tumor ≥1 cm, as measured by CT scans taken after six cycles of second-line chemotherapy, was a poor prognostic factor for OS but not for PFS. Consistent results were also observed in a subgroup of patients with PSR. We recognize that these findings might originate from a small sample size. As this study deals with recurrent EOC rather than primary EOC, we also observed that a considerable number of patients (88.9%, 120/135) experienced relapse despite second-line treatment. Nevertheless, our hypothesis on these results is as follows: despite six cycles of chemotherapy, slow-cycling CSCs might survive within the residual tumor, with a larger size of the residual tumor relating to a greater number of surviving CSCs [22, 23]. Considering the quiescent non-dividing state of CSCs [24], the number of CSCs might not affect the time until progression at this time. However, long-lived CSCs might resist subsequent chemotherapy, resulting in high mortality. Therefore, we believe that slow-cycling CSCs must be effectively eradicated to achieve long-term survival.

Regarding the role of secondary debulking surgery in PSR EOC, recent phase III RCTs reported inconsistent results: while the DESKTOP-III trial showed that secondary surgical cytoreduction followed by chemotherapy significantly elongated patient PFS compared to chemotherapy alone [25], GOG-0213 failed to prove survival benefit from secondary surgical cytoreduction [26]. However, selection criteria and study designs, as well as proportions of complete resection and bevacizumab users, were different between the two studies. Some might argue that patients who achieved complete gross resection from secondary debulking surgery would show better survival outcomes than those who did not. However, to be faithful to the purpose of research, the current study excluded patients who did not have any residual tumor or those who had residual tumor <0.5 cm confirmed via CT scans taken after 6 cycles of second-line chemotherapy. In addition, we conducted additional subgroup analyses by excluding patients who underwent secondary debulking surgery. In the main and subgroup analyses, no survival benefit from extended second-line chemotherapy was observed.

In recurrent EOC, the optimal treatment duration of second-line chemotherapy remains an unanswered issue. Moreover, introduction of new targeted agents, such as bevacizumab and olaparib, is expected to reduce the necessity of additional cycles of conventional chemotherapy. For the treatment of PSR EOC, recent phase III RCTs demonstrated significant PFS gains from maintenance with bevacizumab [9, 10] or olaparib [27]. Similarly, in our study, maintenance therapy with bevacizumab or olaparib was associated with significantly better PFS but not OS. Meanwhile, extended chemotherapy itself did not influence either PFS or OS. However, no comparison was performed according to the type of maintenance therapy because of the small number of patients who received maintenance therapy, especially in the extended group.

Our study has several limitations. First, although this study was a two-institutional retrospective cohort study, the sample size of the study population was small, especially for the extended group (*n* = 52). Thus, there is a chance for selection bias and other statistical issues to exist. Second, although we conducted multivariate analyses adjusting for possible confounders and subgroup analyses according to secondary debulking surgery, issues of study population heterogeneity might not be clearly resolved, which might be answered by well-designed prospective RCTs. Lastly, we did not evaluate toxicities or adverse events, which might have increased with longer treatment periods in the extended group. Nevertheless, we specifically selected our study population by including consecutive patients as long as they met clearly defined eligibility criteria to fairly compare our interests. A similar observation period between the extended and standard groups (median, 33.7 vs. 31.8 months; *P* = 0.617) was also one of the strengths of our study.

## Conclusions

In conclusion, extended chemotherapy might not improve survival outcomes in patients with PSR EOC who showed residual disease after six cycles of second-line chemotherapy. Consistent results were also observed in a subgroup of patients who did not undergo secondary debulking surgery. In the era of maintenance with new targeted agents, we expect the role of extended second-line, platinum-based chemotherapy to be reduced. Further prospective studies are warranted.

## Supplementary Information

**Additional file 1: Figure S1.** Evaluation of residual tumor using CT image. (Upper) A 66-year old woman with recurrent high-grade serous ovarian carcinoma in the extended group. (A) Despite 6 cycles of paclitaxel-carboplatin, she had multiple residual tumors of variable sizes; (B) Stable disease after additional 3 cycles of paclitaxel-carboplatin; (C) New lesions suggesting disease progression. (Lower) A 41-year old women with recurrent low-grade serous ovarian carcinoma in the standard group. (D) Despite 6 cycles of paclitaxel-carboplatin-bevacizumab, multiple peritoneal and mesenteric seeding nodules and residual tumor of the vaginal stump tumor were still observed; (E) Disease progression after 6 cycles of bevacizumab maintenance therapy.

**Additional file 2: Table S1.** Treatment administration in study population.

## Data Availability

The datasets used and/or analyzed during the current study are available from the corresponding author on reasonable request.
