# Supplementary figures and images for: Survival impact of extended cycles of second-line chemotherapy in platinum-sensitive relapsed ovarian cancer patients with residual tumor after six cycles
Source: BMC Cancer. 2020 Dec 7;20:1199. doi: 10.1186/s12885-020-07658-8 (PMC7720565; doi:10.1186/s12885-020-07658-8)

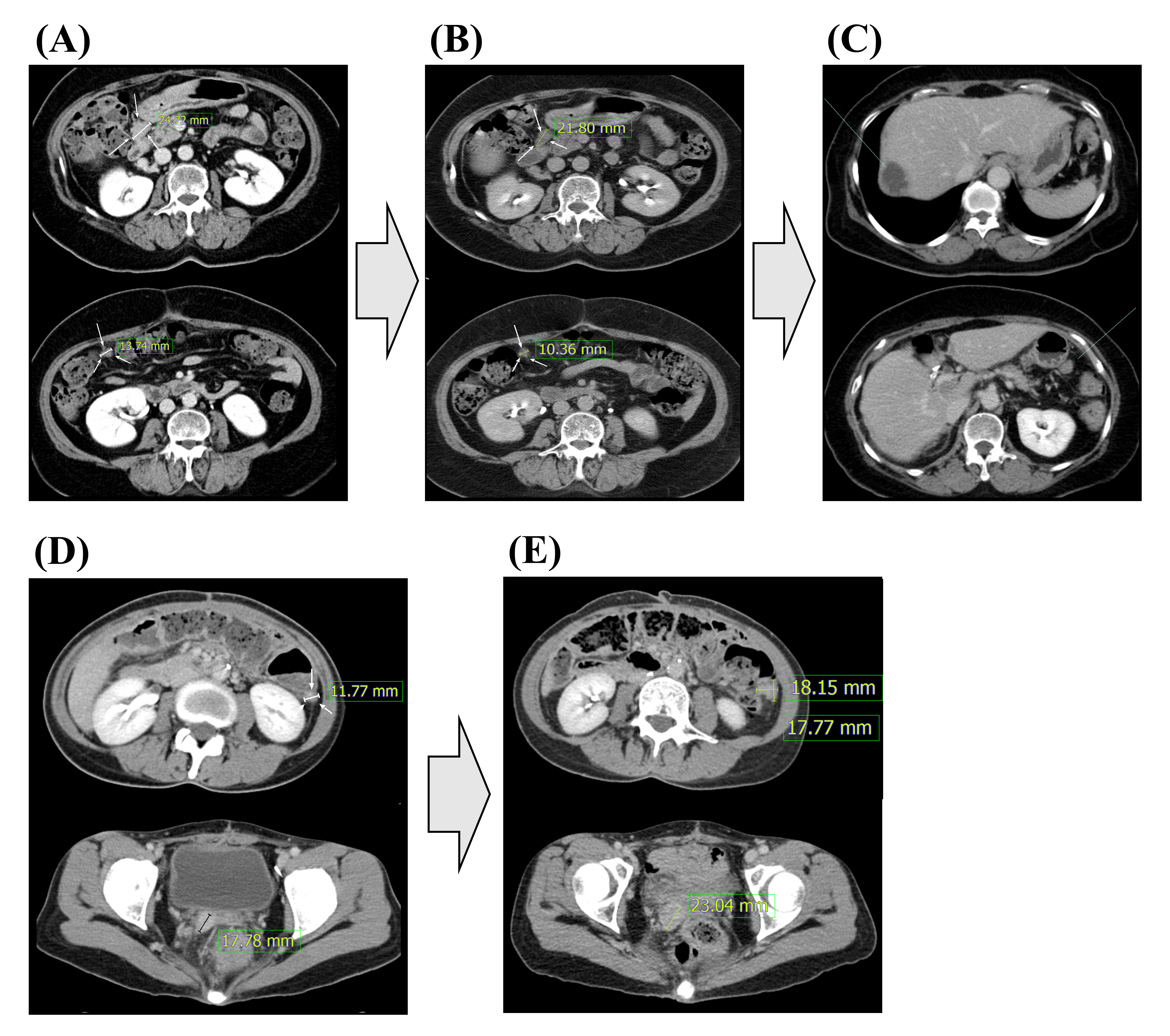

Supplement: Supplementary file 1 — Additional file 1: Figure S1. Evaluation of residual tumor using CT image. (Upper) A 66-year old woman with recurrent high-grade serous ovarian carcinoma in the extended group. (A) Despite 6 cycles of paclitaxel-carboplatin, she had multiple residual tumors of variable sizes; (B) Stable disease after additional 3 cycles of paclitaxel-carboplatin; (C) New lesions suggesting disease progression. (Lower) A 41-year old women with recurrent low-grade serous ovarian carcinoma in the standard group. (D) Despite 6 cycles of paclitaxel-carboplatin-bevacizumab, multiple peritoneal and mesenteric seeding nodules and residual tumor of the vaginal stump tumor were still observed; (E) Disease progression after 6 cycles of bevacizumab maintenance therapy. [file 12885_2020_7658_MOESM1_ESM.png]
