# Supplementary material for: Survival impact of extended cycles of second-line chemotherapy in platinum-sensitive relapsed ovarian cancer patients with residual tumor after six cycles
Source: BMC Cancer. 2020 Dec 7;20:1199. doi: 10.1186/s12885-020-07658-8 (PMC7720565; doi:10.1186/s12885-020-07658-8)
Supplement: Supplementary file 2 — Additional file 2: Table S1. Treatment administration in study population. [file 12885_2020_7658_MOESM2_ESM.docx]

| **Table S1.** Treatment administration in study population | | |  |
| --- | --- | --- | --- |
| Characteristics | *Extended chemotherapy* (*n*=52) | *Standard chemotherapy* (*n*=83) | |
| ***Secondary debulking surgery + 2^nd^ line chemotherapy (n=17)*** |  |  | |
| #6 |  |  | |
| Paclitaxel-Carboplatin #6 |  | 5 (6.0) | |
| Paclitaxel-Carboplatin-Bev #6 |  | 2 (2.4) | |
| Paclitaxel-Carboplatin-Bev #6 + Bev maintenance |  | 6 (7.2) | |
| Paclitaxel-Carboplatin #6 + Olaparib maintenance |  | 1 (1.2) | |
| PLD-Carboplatin #6 + Olaparib maintenance |  | 1 (1.2) | |
| Belotecan-Cisplatin #6 + Olaparib maintenance |  | 1 (1.2) | |
| #9 |  |  | |
| Paclitaxel-Carboplatin-Bev #9 | 1 (1.9) |  | |
| ***2^nd^ line chemotherapy only (n=118)*** |  |  | |
| #6 |  |  | |
| Paclitaxel-Carboplatin #6 |  | 27 (32.5) | |
| Paclitaxel-Carboplatin-Bev #6 |  | 1 (1.2) | |
| Paclitaxel-Carboplatin-Bev #6 + Bev maintenance |  | 15 (18.1) | |
| Paclitaxel-Carboplatin #6 + Olaparib maintenance |  | 2 (2.4) | |
| PLD-Carboplatin #6 |  | 9 (10.8) | |
| PLD-Carboplatin #6 + Olaparib maintenance |  | 2 (2.4) | |
| Gemcitabine-Carboplatin #6 |  | 2 (2.4) | |
| Gemcitabine-Carboplatin-Bev #6 |  | 1 (1.2) | |
| Gemcitabine-Carboplatin-Bev #6 + Bev maintenance |  | 3 (3.6) | |
| Docetaxel-Carboplatin #6 |  | 2 (2.4) | |
| Topotecan-Cisplatin #6 |  | 3 (3.6) | |
| #7 |  |  | |
| Paclitaxel-Carboplatin-Bev #7 | 1 (1.9) |  | |
| PLD-Carboplatin #7 | 1 (1.9) |  | |
| Gemcitabine-Carboplatin #7 | 1 (1.9) |  | |
| Belotecan-Cisplatin #7 | 1 (1.9) |  | |
| #8 |  |  | |
| Paclitaxel-Carboplatin #8 | 3 (5.8) |  | |
| Paclitaxel-Carboplatin-Bev #8 + Bev maintenance | 4 (7.7) |  | |
| PLD-Carboplatin #8 | 1 (1.9) |  | |
| Docetaxel-Carboplatin #8 | 1 (1.9) |  | |
| #9 |  |  | |
| Paclitaxel-Carboplatin #9 | 15 (28.8) |  | |
| Paclitaxel-Carboplatin-Bev #9 | 1 (1.9) |  | |
| PLD-Carboplatin #9 | 1 (1.9) |  | |
| Gemcitabine-Carboplatin #9 | 4 (7.7) |  | |
| Gemcitabine-Carboplatin-Bev #9 | 1 (1.9) |  | |
| Gemcitabine-Carboplatin-Bev #9 + Bev maintenance | 1 (1.9) |  | |
| Docetaxel-Carboplatin #9 | 2 (3.8) |  | |
| Belotecan-Cisplatin #9 | 2 (3.8) |  | |
| Belotecan-Cisplatin #9 + Olaparib maintenance | 1 (1.9) |  | |
| Topotecan-Cisplatin #9 | 1 (1.9) |  | |
| #10 |  |  | |
| Belotecan-Cisplatin #10 | 1 (1.9) |  | |
| #12 |  |  | |
| Paclitaxel-Carboplatin #12 | 3 (5.8) |  | |
| Gemcitabine-Carboplatin #12 | 1 (1.9) |  | |
| #13 |  |  | |
| Belotecan-Cisplatin #13 | 1 (1.9) |  | |
| #15 |  |  | |
| Paclitaxel-Carboplatin #15 | 2 (3.8) |  | |
| PLD-Carboplatin #15 | 1 (1.9) |  | |
| Values are presented as n (%). Abbreviations: Bev, bevacizumab; PLD, pegylated liposomal doxorubicin. | | | |
